# Deep Learning Insights into Lanthanides Complexation Chemistry

**DOI:** 10.3390/molecules26113237

**Published:** 2021-05-27

**Authors:** Artem A. Mitrofanov, Petr I. Matveev, Kristina V. Yakubova, Alexandru Korotcov, Boris Sattarov, Valery Tkachenko, Stepan N. Kalmykov

**Affiliations:** 1 Department of Chemistry, Lomonosov Moscow State University, Leninskie Gory, 1 bld.3, 119991 Moscow, Russia; petrimatveev@gmail.com (P.I.M.); stepan@radio.chem.msu.ru (S.N.K.); 2 Science Data Software, 14909 Forest Landing Cir, Rockville, MD 20850, USA; cubovaya@yandex.ru (K.V.Y.); akorotcov@yahoo.com (A.K.); brois1@mail.ru (B.S.); tkachenko.valery@gmail.com (V.T.)

**Keywords:** lanthanides, deep learning, complexation

## Abstract

Modern structure–property models are widely used in chemistry; however, in many cases, they are still a kind of a “black box” where there is no clear path from molecule structure to target property. Here we present an example of deep learning usage not only to build a model but also to determine key structural fragments of ligands influencing metal complexation. We have a series of chemically similar lanthanide ions, and we have collected data on complexes’ stability, built models, predicting stability constants and decoded the models to obtain key fragments responsible for complexation efficiency. The results are in good correlation with the experimental ones, as well as modern theories of complexation. It was shown that the main influence on the constants had a mutual location of the binding centers.

## 1. Introduction

Processes of metal ions complexation with organic ligands are among the most widespread types of chemical processes [1]. Complexes formation is not only an interesting process itself but also a representative model for the development of sensors and novel pharmaceuticals or chromatography column separation process. Design of the novel compounds with a given binding efficiency for a certain group of metal cations or particular ion selectivity in the presence of other metals is a complex task of molecular recognition. This problem is essential for such practical areas as hydrometallurgy [2], sensing the hazardous metals in the environment [3], metal-containing pharmaceuticals production [4] and metals toxicology [5].

Of course, the approach to the prediction and development of new ligands with specified properties should be based on theoretical foundations. From the theoretical point of view, our understanding of the selectivity of metal-binding processes was based on Pearson’s HSAB theory [6,7,8], and then, together with the development of density functional theory [9,10], it has been transformed into a detailed description of the quantum chemical parameters of the “free ligand-metal-complex” system. Unfortunately, this approach requires large computational resources and results in calculation time comparable to the experimental one.

A fundamentally different approach to predicting properties has machine learning. Though the idea of machine learning, neural networks and “deep learning” is rather old [11], its popularity has significantly increased during the last decade due to the availability of a vast amount of labelled data and optimization of computational costs brought by recent accomplishments in graphics processing unit (GPU) computing [12]. Despite this, the machine learning methods in chemistry are still mostly drug-design-oriented [13]. A number of deep learning applications in chemistry significantly increased in the last few years and now includes topics from a quantitative structure–activity relationship (QSAR) and structure–property relationship (QSPR) to quantum chemistry and materials design [14]. However, machine learning techniques, especially deep learning approaches, are not widely used for predicting metal ions complexation, particularly for purposes of chemical technology. Varnek et al. [15] demonstrated utilization of machine learning approach for Am/Eu separation factor (SF) modeling, using six types of ligand structures. Eventually, this work was developed into “ISIDA Predictor” software [16] currently available online. Moreover, a similar problem was also investigated by Chagnes et al. [17] and Salahinejad and Zolfonoun [18] also on a narrow range of compound classes. Another disadvantage of these approaches is the inability to make a reverse prediction—to show which parts of the molecules influenced the property.

Here we chose binding constants as an important fundamental parameter of metal complexation and examined several types of molecular vectorization algorithms and neural network architectures to build prediction models based on a relatively small number of experimentally determined binding constants. We investigated lanthanides(III) complexation to test our approach on one of the most challenging chemical complexation tasks due to the separation difficulties [19,20,21] and the lack of a sufficient amount of existing experimental data.

**Figure 1:**
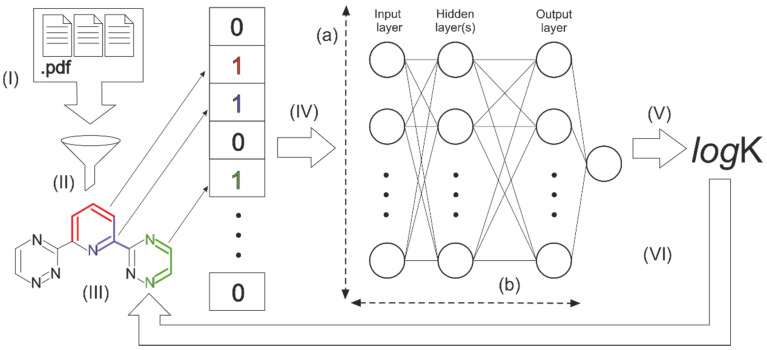
The general scheme of the investigation process.

The study design and machine learning workflow of this work are presented in Figure 1. At the first stage (I), we collected the literature data on the metal ions complexation. All the data were carefully processed (II) to get datasets suitable for modeling and model testing. We encoded molecular structures as digital vectors (III) and investigated several neural network architectures. The neural network was tuned (IV) to train the model showing the best determination coefficient (R^2^) while predicting stability constant (logK) values for the molecules excluded from the training process (test set) (V). Then we used trained models for the reverse process to determine the contribution of molecule fragments (VI). Below you can find a more detailed description of each step.

## 2. Materials and Methods

### 2.1. Datasets

In the presented work we have analyzed from 82 to 324 stability constants for each lanthanide (III) complex stability according to the available complexation data in the literature (25 May 2020). For each metal, the greatest number of constants was observed for complexes with stoichiometry 1:1; however, we also collected data for complexes with two ligands and one metal ion (Table 1). The quality of the data can significantly affect the models quality and predictive ability of the neural network (R^2^ and root mean squared error (RMSE)). Though the total number of constants was essentially small, we selected constants, guided by the following criteria:

- Regardless of the method, we considered the constants obtained only at the ionic strength of 0.1–0.3 M and at temperatures of 20–25 °C.
- In cases of constants obtained in non-aqueous solutions or mixtures with water, we selected only the constants confirmed (coinciding within the error limits) by another independent method.
- In the case of different values of the binding constants for the same complex that were established by different methods, the preference was given to constants measured with the potentiometric titration. It is worth noting that most of the constants in the datasets collected for this study were obtained by this method.
- If the values of the constants obtained by one method and under the same conditions differed greatly from each other, we chose a constant from the work, where the experimental conditions were described in a more accurate way.

The main classes of ligands were carboxylic acids (m.w. from 46 to 174), aminopolycarboxylic (m.w. from 119 to 641) acids and neutral donor ligands with N or O binding centers. Specially designed ligands with high values of stability constants are presented in datasets, as well as ligands with relatively small complexation strength.

**Figure 2:**
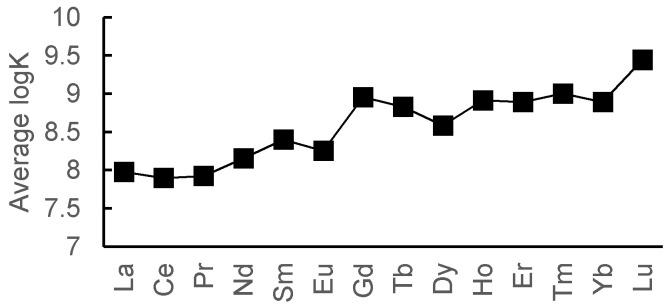
Average logK values for 1:1 complexes.

We calculated the average values of the constants for the purpose of characterization of the datasets (Figure 2). One can see that the average value increases from lanthanum to lutetium. The resulting trend is consistent with the HSAB theory (heavy lanthanides are “harder” than light ones, hence they have higher binging efficiency towards N,O-containing ligands), which means that the collected datasets are representative in terms of lanthanides complexation. The 1:1 datasets are also available in the Supplementary Materials.

### 2.2. Machine Learning

Initial datasets were split into test and training sets with 80% of the molecules used for model training and 20% for model testing. A “stratified” splitting method was used to achieve similar distributions of the endpoint values over train and test datasets. All the calculations were performed by using 10-fold cross-validation. Briefly, after the train–test split, the training set was shuffled and partitioned; thus, 90% of the train test was used for training and 10% were left for validating training steps. In total, 10 sub-models were trained and validated. Finally, we averaged results over the models to get a single answer for each ligand–metal pair. The quality of the models was evaluated by using a coefficient of determination, R^2^, and root mean squared error over concurrence of predicted and actual values for validation and test datasets.

Neural networks were built and trained, using an open-source TensorFlow library (version 1.4.1) [22] with Keras (version 2.1.3) application programming interface [23]. The distribution of weights of network nodes was limited by l2 regularization. We also applied drop-out for the hidden layers. Scikit-learn library was used for train/test splitting, cross-validation, shuffling and data scaling using MinMax algorithm. All the scripts were written in Python language. Cheminformatics library RDKit [24] was used for structures validation, standardization and descriptors generation.

### 2.3. Molecule Fingerprints

**Figure 3:**
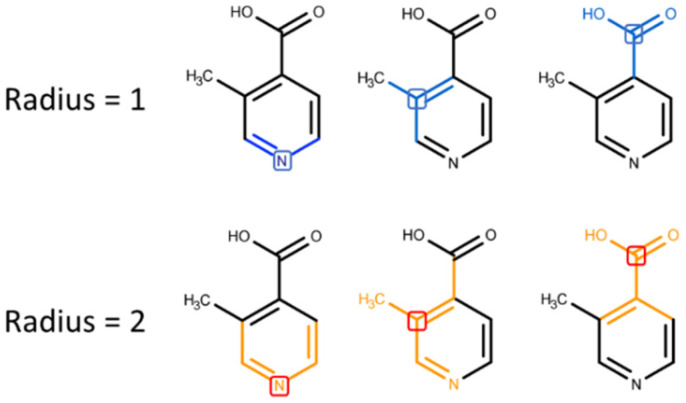
Molecular fragments encoded in FCFC fingerprints with different radii.

Digital molecule representation is probably the most important question of the model building process. The way used to transform a molecule into a row of digits determines information that will be used for model training. There are a lot of algorithms developed for molecular representation. Here we used the popular Morgan [25] algorithm for generating so-called circular fingerprints of the molecules. Circular fingerprints, in general, is a way to represent molecule structure by using the information of atoms’ neighborhoods. Circular fingerprints (FCFC) follow the route of encoding information rather than the calculation of predefined features and can capture a large amount of the local structural information that is present in a molecule. It works by applying the Morgan algorithm to a set of predefined atom invariants, making it similar to a convolution operation over a graph. Connectivity-based invariants, such as element, number of heavy neighbors, number of hydrogens, charge, isotope, and in ring or out, use molecules’ connectivity information similar to the way it is done in ECFP-type fingerprints. Moreover, chemical-features-based invariants are available: Donor, Acceptor, Aromatic, Halogen, Basic and Acidic. FCFC descriptors have a large applicability domain expressed in good performance over a notable range of datasets and endpoints described in a number of articles [26,27,28]; thus, they were chosen for further development (Figure 3).

**Figure 4:**
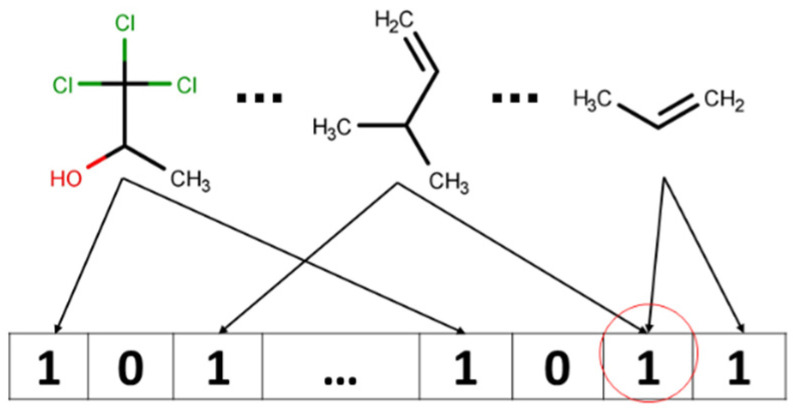
Possible bit collision: information about two fragments is encoded into the same bit.

Due to the way in which Morgan Fingerprints are folded to be a particular size (…, 512, 1024, 2048,…), several different bits (corresponding to two different atom environments) can be written to the same bit; when this occurs, it is usually referred to as collision [25,26] (Figure 4).

Collisions can cause ambiguity when analysis and interpretation of the molecule’s bitstring are attempted. In this study, we performed feature importance analysis to find out which features in the input feature vector (or which fragments of the molecule) have the biggest impact on the end-point value (Y). However, it might be difficult to distinguish which particular submolecular pattern corresponds to the feature when it is activated by several bits in the case of when bitstring representation was hashed by using Morgan fingerprints.

Increasing fingerprint length decreases the number of colliding bits [29] but also significantly increases computational time, so we used 256-bit fingerprints and took only non-colliding bits for further analysis. Moreover, we used a radius equal to 2 as the most interpretable from the general chemical sense.

## 3. Results and Discussion

### 3.1. Models

We have built several neural network architectures with a different number of nodes in a layer (from 256 to 4096) and a number of layers (from 1 to 5), marked as (a) and (b) correspondingly in Figure 1. Growth of the parameters significantly increased the time of the network training process but did not improve the models’ performance as much. We chose a three-layer network with 512 nodes in a layer as the best ratio of time and efficiency.

### 3.2. Models Accuracy

**Figure 5:**
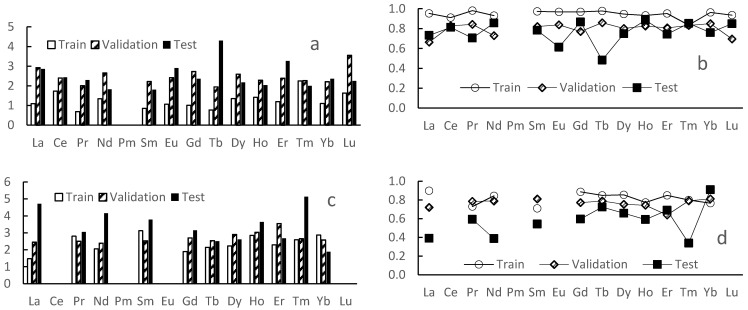
Performance of trained models: root mean squared error (**a**,**c**) and coefficient of determination (**b**,**d**) for 1:1 (**a**,**b**) and 1:2 (**c**,**d**) complexes.

The models’ efficiency parameters are presented in Figure 5. The mean determination coefficient value on test sets for all the 1:1 complexation models is about 0.76. Relatively high (comparable with “test” ones) values of the coefficient for validation sets approve independence of the models’ performance on the train/test separation process.

On the other hand, the lack of data did not allow us to build the same models for the most of 1:2 complexes as also shown in Figure 5 where R^2^ values are significantly smaller.

The mean RMSE value for 1:1 complexes is 2.5, which seems to be too big for a purely quantitative method, but it is enough for estimation of ligands separation efficiency.

### 3.3. Fragment Importance Analysis

Trained models can be used for predicting stability constants, but they do not explain the chemistry of the complexation process and fundamentals of ligands selectivity, i.e., also important for new ligands design.

While simple machine learning methods, such as linear regression, are easily interpretable, the more complex (and, at the same time, more accurate) seem to be a kind of “black-box” tool generating answers without any chemical basement. Here, we used SHAP (SHapley Additive exPlanations) approach [30] for sensitivity analysis and refinement of our “structure–property relationship” up to determining contributions of molecule parts and functional groups to the target property. It can estimate the contribution (either positive or negative) of each bit in the initial vector to the final value predicted by the model. The set of SHAP values can be calculated for each vector (each molecule) separately, and the approach can be used in combination with any kind of machine learning method. The algorithm is based on variating components of the input vector in the model train set vector space and unifies a number of methods described in the literature [31,32,33].

In order to decrease the computational time, we narrowed the train sets’ vector space up to 30 representatives via “k-means” algorithm [34,35] based on minimizing total within-cluster Euclidian distance to cluster centroid (Equation (1)):

(1) $$\overset{N}{\underset{i=0}{\sum }}\underset{\mu_{j}\in C}{\min}\left({\left\|x_{j}-\mu_{i}\right\|}^{2}\right)$$

Thus, we investigated each vector of the train set with the variational approach in a space formed by 30 other vectors represented entire train set and received a set of “contribution vectors” as a result. At first, we discarded zero values that corresponded to absence of the fragment. We calculated mean and RMSE for all the rest non-zero values and again discarded those where RMSE was bigger than the mean by absolute value. Finally, we believe in concurrences; thus, we left only values describing fragments presented in more than two molecules of the train set.

**Figure 6:**
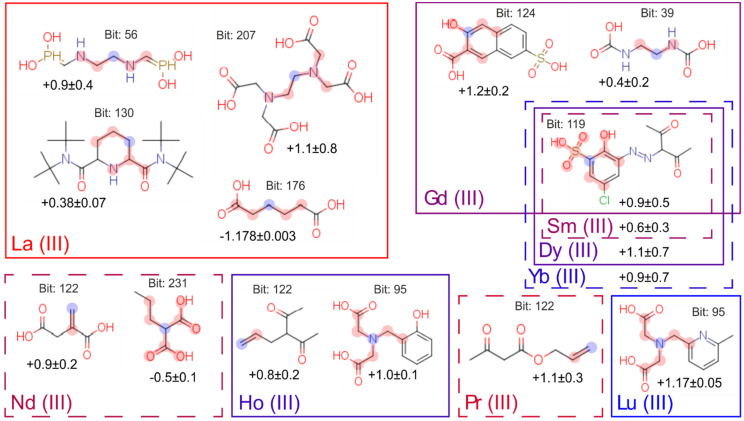
Fragments of molecules significant for Ln(III) complexation. Fragments are signed with red (blue for central atom according to Morgan algorithm); values of additive impact to stability constant are undersigned for each metal.

After the calculations, we got the results, and they are presented in Figure 6. The numbers under the molecules describe the mean contribution of fragments to logK value. For example, according to the model, adding terminal double bond to ligand will increase the logarithm of its complex stability constant with Pr(III) by 1.1.

We compared the results with well-known trends in lanthanides complexation, and we can interpret them as follows:

- The group containing soft aromatic nitrogen appears only for lanthanum cation. This is consistent with the fact that La(III) is the softest acid in the lanthanide series in terms of Pearson’s theory [8].
- From the point of view of the relative location of the binding centers, we can note that the structural fragments resulting in the formation of the most stable five-membered cycles [36] (bits: 56, 207, 39 and 95) lead to an increase in the value of the constant, and, in contrast, the long alkyl chain (bit: 176) between two carboxylic acids leads to decrease of stability constants. For the second group with the negative influence on constant’s value (bit: 231), we can admit formal match—propylmalonic acid includes a four-carbon-atoms chain (with carboxylic group). On the other hand, a six-membered cycle of malonic acid is less stable than a five-membered one [37]. Thus, we can conclude that this bit is also emphasizing the importance of the relative location of the binding centers.
- For two cations, Ho(III) and Lu(III), bit 95 has a positive contribution in binding, which corresponds to ethyl dicarboxylic acid amine. It is well-known [38] that, for all polyaminocarboxylates, the binding constant increases with the increase of the atomic number in the lanthanide series. Thus, this fragment should influence binding with all lanthanides. Perhaps the impact of this bit on Ho(III) and Lu(III) is due to the fact that these cations are one of the hardest acids in the lanthanide series.
- In the case of Sm(III), Gd(III), Dy(III) and Yb(III), rigid phenyl ring with two neighboring binding groups (bits 119 and 224) affects binding positively. In this case, the formation of six-membered cycles also occurs. We can also note that the presence of binding centers on the phenyl ring leads to their preorganization, which eventually increases the binding constant [39].
- Unexpectedly, the contribution to the binding is made by bit corresponding to double C=C bonds (bit 122). Compounds with Ln(III)–C=C interaction are known as catalysts [40,41], but these groups cannot be used as binding sites for the development of water-soluble complexions.

It is also interesting to notice that typical binding centers are not in a list of the most important fragments. It can be easily explained by the fact that they were presented in all the molecules of collected datasets, and their contribution is nearly the same for all the complexes.

## 4. Conclusions

In conclusion, we have the shown possibility of training predictive models by using relatively small collections of chemical data (comparing with thousands in common datasets in cheminformatics) and simple neural network architectures. We believe that the models for Lns(III) complexation can be used for a semi-quantitative estimation of complexation constant values at the very first stage of complexing agent design (with the mean uncertainty equal to ~2.5 [logK]). We have also shown a way of computational analysis of a machine learning model that can be expressed in a common chemical way and may lead to a deeper understanding of the physical or chemical driving force of the modeled process. Thus, according to the model analyses, the main influence on the value of the lanthanide binding constant is the mutual arrangement of the binding centers, such as N-/O- donors. Of course, though this work is devoted to lanthanide chemistry and neural networks, the proposed approach is insensitive to the machine learning algorithm and can be easily broadened to other metals or other QSPR models.

## Figures and Tables

**Table 1:** Amounts of molecules in the collected datasets.

Me(III)	No. Molecules (L1:Me1/L2:Me1)	Me(III)	No. Molecules (L1:Me1/L2:Me1)
La	236/88	Tb	177/74
Ce	82/-	Dy	161/73
Pr	151/65	Ho	163/69
Nd	192/82	Er	138/74
Sm	138/75	Tm	149/66
Eu	224/-	Yb	212/67
Gd	156/96	Lu	236/80

## Data Availability

The datasets are available in the Supplementary Materials. The software is available on https://github.com/scidatasoft/ml-services (accessed on 25 May 2021).

## Supplementary Materials

The following are available online, 1:1 datasets.
